# *TMEM230* in Parkinson’s disease in a southern Spanish population

**DOI:** 10.1371/journal.pone.0197271

**Published:** 2018-05-17

**Authors:** Cristina Tejera-Parrado, Silvia Jesús, Adrián López-Ruíz, Dolores Buiza-Rueda, Marta Bonilla-Toribio, Inmaculada Bernal-Bernal, María Teresa Periñán, Laura Vargas-González, Pilar Gómez-Garre, Pablo Mir

**Affiliations:** 1 Unidad de Trastornos del Movimiento, Servicio de Neurología y Neurofisiología Clínica, Instituto de Biomedicina de Sevilla (IBiS), Hospital Universitario Virgen del Rocío/CSIC/Universidad de Sevilla, Sevilla, España; 2 Centro de Investigación Biomédica en Red sobre Enfermedades Neurodegenerativas (CIBERNED), Madrid, España; University College London Institute of Neurology, UNITED KINGDOM

## Abstract

*TMEM230* has been associated with autosomal dominant Parkinson’s disease (PD). Subsequent studies have remained negative, and none of previous described mutation has been reported anymore. We investigated the implication of this gene in the PD in a population of 703 PD patients and 695 unrelated healthy controls from southern Spain. Thirteen variants were found, twelve of them observed only in controls or in patients and controls, and one (c.190A>G) observed only in one patient. Subsequent analysis of this variant indicates that probably it is not pathogenic. In addition, we found a variation in the 3’-UTR (rs183551373) and related with the miRNA hsa-miR-4299 but it was observed only in healthy controls. Our results suggest that variants in *TMEM230* gene are not associated with the development of PD.

## Introduction

Parkinson’s disease (PD) is a complex pathology in which both environmental and genetic factors are involved. More than 18 genes, like *PARK2*, *LRRK2* or *SNCA*, among others, are well documented as causative genes or risk factors for PD [1]. However, the study of other described genes is still needed for understanding their implication in the pathophysiology of PD.

Recently, a missense mutation (p.Arg141Leu) in *TMEM230* gene has been described to be a cause of autosomal dominant PD in a large family from North American [2]. Two other mutations (p.Tyr92Cys and p.*184Trpext5*) were also identified in two young onset unrelated PD patients in that study. Furthermore, in a Chinese population, a new mutation in *TMEM230* (p.*184ProGlyext5*) was identified as being related with PD in 7 familial cases. Several additional studies in different populations have failed to find neither causative mutation nor risk factors in *TMEM230* associated with PD [3–16] (S1 Table).

In order to complete the picture of the involvement of *TMEM230* gene in PD, we have analyzed this gene in a PD population from southern Spain.

## Materials and methods

The study was approved by the Ethics Committee of the Hospital Virgen del Rocío and was conducted according to the Declaration of Helsinki. All subjects, both patients and controls, were informed (orally and in writing) of the nature of the study and signed a written consent before blood collection.

We included a total of 703 PD patients from the Movement Disorders Unit of the Hospital Universitario Virgen del Rocío (Seville, Spain), and 695 healthy controls (HC). One hundred forty-eight patients had familial PD (Table 1). PD was diagnosed using the criteria of the United Kingdom Parkinson’s Disease Society Brain Bank [17]. A positive family history was defined as the presence of Parkinson’s disease in at least other first-degree and/or second-degree relative. Signed consent forms were obtained for each individual studied and the study was approved by the local ethic committee. Genomic DNA was isolated from peripheral blood from each subject, according to established protocols by standard or automated methods (MagNA Pure LC, Roche Diagnostics, Indianapolis, IN). To detect mutations, all the 5 exons of *TMEM230* (NM_001009923 and NM_001009925) and their intron boundary regions were analyzed using high-resolution melting analysis in a LightCycler 480-II (Roche). Samples showing abnormal melting profiles were sequenced in an ABI3500 Genetic Analyzer (Applied Biosystem). The identified variants were annotated according to the longest isoform (NM_001009923). A variant call of sequenced samples was made using Variant Reporter software v1.1. (Applied Biosystem).

**Table 1:** Demographic characteristics of studied population.

Subjects (n)		% males (M/F)	Mean age (y)	Mean AO (y)
Healthy controls (695)		55. 4 (385 / 310)	57 ± 15	-
PD	Total (703)	54.6 (384 / 319)	64 ± 12	56 ± 13
	Familial (148)	45. 9 (68 / 80)	62.5 ± 11	53 ± 14

PD: Parkinson’s disease; y: years; AO: age at onset; n: number of samples

## Results

We detected a total of 13 genetic variants (two not reported before) in our population: two at 3’-untranslated region, one in 5’-untranslated region, one intronic variant, four synonymous variants, and five nonsynonymous variants (Table 2). Ten were rare variants (minor allele frequency <0.01). From those, four variants were observed in patients and controls (rs141394228, rs147693982, rs148033002, and c.*670T>C) and five were observed only in controls (rs368686615, rs186628284, rs776037148, rs143571424, and rs183551373). One novel variant (c.190A>G) was present in one patient only. This variant results in the loss of the start codon in the most common isoform of *TMEM230*. However, another variant found in the present study, c.191T>C, carrying the same effect was present in two healthy controls and in one PD patient. Another novel variant was identified in 3’UTR region, c.*670T>C but it was present in patients and control subjects. None of these variations were identified in the familial cases. Neither of the known variants previously related with PD have been found in this study.

Three frequent variants (rs10221980, rs6107576, and rs6116651) were present in PD case cohort and control cohort with similar frequencies.

**Table 2:** Variants identified in *TMEM230* in our population.

Chromosome position ^&^	Variant^§^			Maf			PD population (Pres/Abs)
	Nucleotide change	Aminoacid change	rs number	1000 genomes^#^	ExAc	ESP	
Chr20: 5093685	c.-11C>T	-	rs368686615	NI	0	0	Abs
Chr20: 5092233	c.87G>A	p.Ser29Ser	rs186628284	NI	NI	NI	Abs
Chr20: 5092141	c.174+5G>C	-	rs10221980	0.103	0.3214	NI	Pres
Chr20: 5090076	c.190A>G	p.Met64Val	-	NI	NI	NI	Pres
Chr20: 5090075	c.191T>C	p.Met64Thr	rs141394228	0.004	0.0018	0.0015	Pres
Chr20: 5090057	c.209A>G	p.Asn70Ser	rs776037148	NI	1.5 e-05	NI	Abs
Chr20: 5086939	c.306T>A	p.Pro102Pro	rs6116651	0.0905	0.1114	0.1093	Pres
Chr20: 5086918	c.327C>T	p.Ile109Ile	rs147693982	0.0169	0.0162	0.0181	Pres
Chr20: 5086915	c.330A>G	p.Ala110Ala	rs6107576	0.0099	0.0126	0.0136	Pres
Chr20: 5086870	c.375A>G	p.Ile125Met	rs148033002	0.001	0.0012	0.0008	Pres
Chr20: 5081478	c.511C>T	p.Arg171Cys	rs143571424	0	0.0039	0.0020	Abs
Chr20: 5080819	c.*618A>G	-	rs183551373	0	0	NI	Abs
Chr20: 5079983	c.*670T>C		-	NI	NI	NI	Pres

Chr: chromosome; &: GRCh37/hg19)

§: referred to the longest *TMEM230* isoform (NM_001009923, NP_001009923)

#: European population

*ExAC: Exome Aggregation Consortium (Non-Finnish European)

ESP: Exome Sequencing Project (European American); NI: No information. Pres/Abs: present or absent in our analyzed PD population

## Discussion

In this study we have reported 13 variants in *TMEM230* but neither of them seems to be related with the development of PD, since they are present only in HC or in both patients and HC. The exception has been c.190A>G, which is present in a single patient. This variation causes the loss of the ATG start codon in the isoform 2 of *TMEM230* (NP_001009925; c.1A>G, p.Met1Val), therefore, it could be disease causing. However, the variant c.191T>C, with similar consequences in the protein (NP_001009925; c.2A>G, p.M1Thr), is present in healthy controls in this study and in previous ones [3]. In addition, the subsequent amino acid of *TMEM230* protein is also methionine, which would act as an alternative start codon to generate a novel protein that lacks only the first amino acid. So, it is expected that this variation does not cause a significant alteration of the protein. In addition, a variation in the 3’-UTR (rs183551373) has been related with the miRNA hsa-miR-4299 but it was found only in healthy controls in our population and, thereupon, the pathogenicity of this variant seems not be probable.

## Conclusions

Our findings suggest that the incidence of pathogenic variations in *TMEM230* is very low and, therefore, *TMEM230* do not play a major role in familial and sporadic PD patients in southern Spanish population which can have important implication in clinical investigation.

## Supporting information

S1 Table Summary of characteristics of previously reported studies for TMEM230 (up to April 2018). (PDF)
