# Supplementary material for: TMEM230 in Parkinson’s disease in a southern Spanish population
Source: PLoS One. 2018 May 17;13(5):e0197271. doi: 10.1371/journal.pone.0197271 (PMC5957438; doi:10.1371/journal.pone.0197271)
Supplement: S1 Table — (PDF) [file pone.0197271.s001.pdf]

Supplementary Table 1. Summary of characteristics of previously reported studies for TMEM230 (up to April 2018).

Reference	Population	N PD (F) / HC	Ancestry	Position (GRCh37; chr20)	Exon	Variation (NM_001009923; NP_001009923)			Frequency		
						Change of Nt	Change of Aa	rs ID	PD	FPD?	HC
Deng et al. 2016	North America	832 (433) /~ 1000	-	5090075	3	c.191T>C	p.Met64Thr	rs141394228	2 / 832	ND	5 / 1081
				5089991	3	c.275A>G	p.Tyr92Cys	rs1056737920	1 / 832	No	0 / 1081
				5086870	4	c.375A>G	p.Ile125Met	rs148033002	2 / 832	ND	3 / 1028
				5081567	5	c.422G>T	p.Arg141Leu	None	1 PD	Yes	0 / 1238
				5081478	5	c.511C>T	p.Arg171Cys	rs143571424	1 / 832	ND	3 / 1238
				5081438	5	c.551A>G	p.*184Trpext*5	-	1 / 832	Yes	0 / 1238
	Chinese	574 (225) /~ 528	-	5081437	5	c.550_552delTAGinsCCCGGG	p.*184ProGlyext*5	-	7 / 574	Yes	0 / 528
Yan et al. 2017	Chinese (western)	1235 (192) / 1252	-	5086888	4	c.357G>A	p.Gly119Gly	rs749201963	1 / 1235	Yes	0 /1252
				5081434	5	c.*3G>T	3'-UTR	-	2 / 1235	No	0 /1252
Giri et al. 2017	Caucasian	1450 / 2267	North America	5086929	4	c.316T>C	p.Tyr106His	rs374122606	1 / 1450	No	0 / 2267
				5081505	5	c.484A>G	p.Ile162val	rs368707598	1 / 1450	No	0 / 2267
		29 (29) / 0	European	5081502	5	c.487G>A	p.Ala163Thr	rs374122606	1[¥] / 29	Yes	#
He et al. 2017[&]	Chinese Han (eastern)	414 (207) / 400	-	5081439	5	Negative for p.*184Trpext*5 (c.551A>G) and p.*184ProGlyext*5 (c.550_552delTAGinsCCCGGG)			0 / 414	-	0 / 400
Baumann et al. 2017	Caucasian	53 (53) / 0	Ukranian	5090063	3	c.203C>T	p.Arg68His	rs780460399	1 / 53	Yes	#
			German	5081478	5	c.511C>T	p.Arg171Cys	rs143571424	1 / 53	Yes	Described by Deng et al
Wu et al. 2017	Chinese (eastern)	122 (122) / 0	-	5093629	1	c.46G>A	p.Glu16Arg	rs1272596579	1[¥] / 122	Yes	NA
				5092135	2	c.174+11C>T	(intronic)	rs144736056	1 / 122	Yes	#
Quadri et al. 2017	Taiwanese[%]	815 (98) / 417	-	5081595	5	c.494A>G	p.Tyr165Cys	rs758033952	1 / 815	No	417[#]
				5081426	5	c.*11A>G	(3'-UTR)	rs750802038	1 / 815	No	417[#]
	Dutch[%]	90 (31) / 0	-	-	5	None			0 / 90	-	NA
	White	266 (266) / 0	Italian	5093674	1	c.1A>G	p.Met1Val	rs768390203	1 / 266	Yes	#
			Brazilian	5092251	2	c.69A>G	p.Arg23Arg	-	1 / 266	Yes	NA
			-	5090075	3	c.191T>C	p.Met64Thr	rs141394228	2 / 266	Yes	Described by Deng et al
				5086870	4	c.375A>G	p.Ile125Met	rs148033002	2 / 266	Yes	Described by Deng et al
				5081478	5	c.511C>T	p.Arg171Cys	rs143571424	2 / 266	Yes	Described by Deng et al
Boungarzone et al. 2017	Caucasian (Italian)	86 (86) / 0	-	5092141	2	c.174+5G>C	(intronic)	rs10221980	3 / 86	Yes	#
				5090075	3	c.191T>C	p.Met64Thr	rs141394228	1 / 86	Yes	Described by Deng et al[#]
				5086939	4	c.306T>A	p.Pro102Pro	rs6116651	13.37%	Yes	#
				5086936	4	c.309G>A	p.Lys103Lys	rs763383477	1 / 86	Yes	#
				5086915	4	c.330A>G	p.Ala110Ala	rs6107576	3 / 87	Yes	#
				5081621	5	c.412-44G>A	(intronic)	rs201276122	2 / 86	Yes	#
Yang et al. 2017	Chinese (southwestern)	366 (11) / 0	-	5093629	1	c.46G>T	p.Gly16Trp	rs1272596579	1 / 366	No	Also described by Wei et al
				5093607	1	c.68G>A	p.Arg23Gln	rs765701142	3 / 366	Yes	Also described by

											Fan et al
				5092253	1	c.69-3T>A	(intronic)	rs1034386774	1 / 366	No	-
				5086963	4	c.289-7C>T	(intronic)	rs767174424	1 / 366	No	#
				5086917	4	c.328G>A	p.Ala110Thr	rs1055543199	2 / 366	No	-
Shi et al. 2017	Chinese Han	550 (0) / 560	-	5093425	1	c.68+182G>A	(intronic)	rs149865687	maf 0.032	No	maf 0.018
				5092242	2	c.78A>G	p.Leu26Leu	rs745443202	3 / 550	No	1 / 560
				5092135	2	c.174+11C>T	(intronic)	rs144736056	MAF 0.0172	No	MAF 0.0143
				5081426	5	c.*11A>G	(3′-UTR)	rs750802038	5 / 550	No	4 / 560
Wei et al. 2018	Chinese Han (southwestern)	120 (120) / 650	-	5093629	1	c.46G>T	p.Gly16Trp	rs1272596579	1 / 120	Yes	0 / 650
				5081560	5	c.429delT	p.Val143ValfsX4	-	0 / 120	-	1 / 650
Ibanez et al. 2017	American European	499 / 294	WUSTL	Negative for those variants previously described by Deng et al					0 / 499	-	0 / 294
		340 / 140	PPMI	5093490	1	c.68+117C>A	(intronic)	rs41282112	ND	ND	ND
				5093410	1	c.68+19C>G	(intronic)	rs41282110			
				5090075	3	c.191T>C	p.Met64Thr	rs141394228	ND	ND	Described by Deng et al
				5086939	4	c.306T>A	p.Pro102Pro	rs6116651	ND	ND	Also described by Boungarzone et al.
				5086935	4	c.310A>C	p.Ile104Leu	rs139824737			#
				5086918	4	c.327C>T	p.Ile109Ile	rs147693982			#
				5086915	4	c.330A>G	p.Ala110Ala	rs6107576			Also described by Boungarzone et al.
				5081478	5	c.511C>T	p.Arg171Cys	rs143571424	ND	ND	Described by Deng et al
				5081276	5	c.*161C>T	(3′-UTR)	rs1249078621	ND	ND	ND
				5081049	5	c.*388A>G	(3′-UTR)	rs2715			
				5080819	5	c.618*A>G	(3′-UTR)	rs183551373			
				5080691	5	c.*746G>A	(3′-UTR)	rs45610034			
				5080631	5	c.*806A>T	(3′-UTR)	-			
				5080560	5	c.*877A>C	(3′-UTR)	rs540970582			
Ma et al. 2017	Chinese (southeastern)	99 (23) / 99	-	5086883	4	c.362T>C	p.Phe121Ser	rs1393260987	0 / 99	-	1 / 99
Fan et al. 2017	Taiwanese	680 (180) / 992	-	5093607	1	c.68G>A	p.Arg23Gln	rs765701142	3 / 680	Yes	3 / 992
				5093573	1	c.68+34C>T	(intronic)	rs999822627	Yes	ND	ND
				5093498	1	c.68+109C>T	(intronic)	rs565123586	Yes	ND	ND
				5093425	1	c.68+182G>A	(intronic)	rs149865687	Yes	ND	Also described by Shi et al
				5092135	2	c.174+11C>T	(intronic)	rs144736056	Yes	ND	Also described by Shi et al
Conedera et al. 2018	Japanese	182 (182) / 0	-	Negative (for coding variations)					0 / 182	-	ND

This table summarizes the data from different studies in which variants in TMEM230 have been tested in relation with Parkinson disease. Articles appear in the same order that they appear in PubMed. ND: No data. Population: it is referred to the origin of the studied population. Ancestry: the ancestry of those PD patients carrying the variations (when the populations are from different countries). N: number of screened samples. Nt: nucleotide. Aa: Amino acid. rs ID: reference SNP ID number PD (F): PD cases (familial cases included). HC: Healthy controls. FPD: Familial PD cases. ¥: This variant does not segregate with PD in the family. #: This variant is present in populations from Exome Aggregation Consortium (ExAC). &: This study is focused in

detect variations affecting to the stop (TAG) codon. %: This study is focused on the study of the exon 5 of the gene. WUSTL: GWASs data and DNA from the Washington University in Saint Louis (This cohort was only analized for those variants previously described by Deng et al). PPMI: GWASs and Whole Exome Sequencing data from the Parkinson's Progression Markers Initiative.
